# P-1021. Comparative Clinical profile, Anti-Fungal resistance rates and in-hospital mortality in hospitalized patients with Candidaemia caused by *Candida Auris* versus Non-Auris Candida in a tertiary-care centre

**DOI:** 10.1093/ofid/ofae631.1211

**Published:** 2025-01-29

**Authors:** Vasant Nagvekar, Anusha Bubna, Shashikala shivaprakash, Akash Shukala, Ravi Mohanka, prashtana rao, Neha Patel, Charudatt Vaity, Bindu Malakavalpil, Rahul Pandit, Preeti Pillai, Omkar Borde, Neetu Mundra

**Affiliations:** Lilavati Hospital / H N Reliance Hospital, mumbai, Maharashtra, India; Hnreliance Hospital, Mumbai, Maharashtra, India; Hnreliance Hospital, Mumbai, Maharashtra, India; Hnreliance Hospital, Mumbai, Maharashtra, India; Hnreliance Hospital, Mumbai, Maharashtra, India; Hnreliance Hospital, Mumbai, Maharashtra, India; Hnreliance Hospital, Mumbai, Maharashtra, India; Hnreliance Hospital, Mumbai, Maharashtra, India; Hnreliance Hospital, Mumbai, Maharashtra, India; Hnreliance Hospital, Mumbai, Maharashtra, India; HN Reliance Hospital, Mumbai, Maharashtra, India; Hnreliance Hospital, Mumbai, Maharashtra, India; Hnreliance Hospital, Mumbai, Maharashtra, India

## Abstract

**Background:**

Candidemia, a severest manifestation of invasive candidiasis, often encountered in intensive care units (ICUs) is predisposed by prolonged ICU-stay, immunosuppression or mechanical ventilation. In India, while various species of Candida (*C. tropicalis*, *C. albicans*, *C. auris* and others) are involved in candidemia, *C. auris* is particularly known for displaying higher anti-fungal resistance. We investigated, whether there was a difference in the clinical pattern of candidemia caused by *C. auris vs* non-auris Candida in our tertiary-care centre.

**Figure 1.**
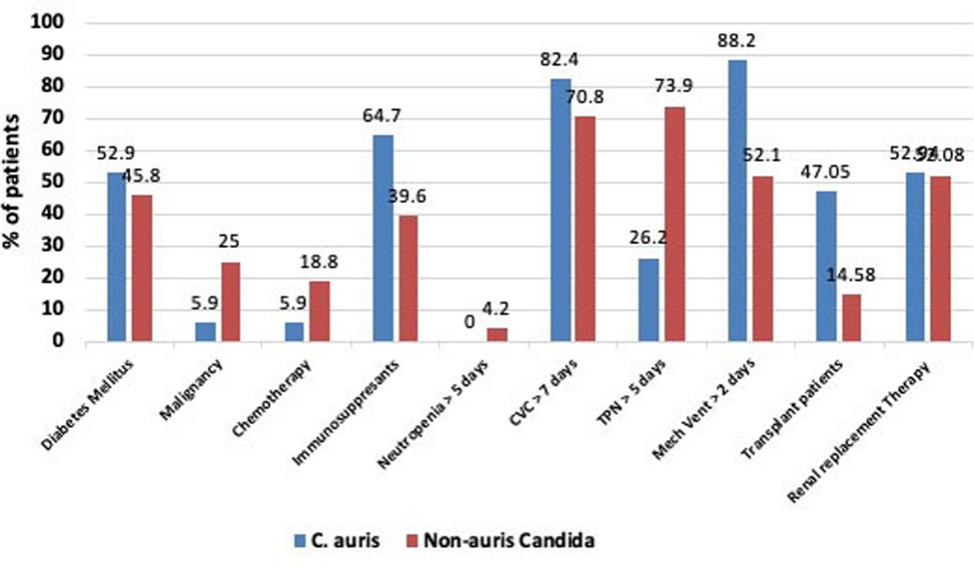
Comorbidities among patients with candidaemia: C. auris group versus non-auris candida group

CANDIDA AURIS VS NON-AURIS CLINICAL RISK FACTORS

**Methods:**

The analysis involved 65 unique episodes (one patient per episode) of documented candidemia in our tertiary-care centre during January 2022 to April 2024. The clinical data was retrieved from the blinded hospital records. The day of admission of patients in to ICU was considered as hospital day-1.

**Results:**

Among 65 episodes, 17 (26.2%) and 48 (73.8%) were that of *C. auris* and non-auris candida, respectively. The time to blood culture positivity was longer with *C. auris* cohort compared to non-auris candida cohort (median hospital days 27 *vs* 19). Total hospitalization days (median) were 49 and 15.5, P value 0.002 respectively. In-hospital mortality was 58% in *C. auris* group and 62.5% in non-auris candida group. There was more proportion of cancer patients in non-auris candida cohort while use of immunosuppressive drugs was more frequent in *C. auris* cohort (Figure 1). Resistance rates to fluconazole, amphotericin, and echinocandins were higher in *C. auris* group (94.5% *vs* 25%; 70.5% *vs* 6.3%; 11% *vs* 2.1%, respectively)..Total Parenteral Nutrition > 5 days (26.2% vs 73.9%) P value 0.38, Transplant Patients (47.05% vs 14.58%) , immunosuppressive drugs (64.7% vs 39.6% ) P value 0.07 and Mechanical Ventilation (88.2% vs 52.1%) P value 0.008 respectively.

**Conclusion:**

*C. Auris* has emerged as a notorious Non Albicans Candida species with substantial morbidity and mortality suggesting change in the epidemiology of candidaemia. Patients with Candida auris had longer hospital stay and blood to positivity than others. Fluconazole resistance remains highest in candida auris than other species of candida and majority of isolates of C.auris had high MICs for amphotericin B.

**Disclosures:**

**All Authors**: No reported disclosures

